# Miniature Resistance Measurement Device for Structural Health Monitoring of Reinforced Concrete Infrastructure

**DOI:** 10.3390/s20154313

**Published:** 2020-08-02

**Authors:** Dean M. Corva, Seyyed Sobhan Hosseini, Frank Collins, Scott D. Adams, Will P. Gates, Abbas Z. Kouzani

**Affiliations:** 1 School of Engineering, Deakin University, Geelong, VIC 3216, Australia; d.corva@deakin.edu.au (D.M.C.); scott.adams@deakin.edu.au (S.D.A.); 2 Institute for Frontier Materials, Deakin University, Burwood, VIC 3125, Australia; sshossei@deakin.edu.au (S.S.H.); frank.collins@deakin.edu.au (F.C.); will.gates@deakin.edu.au (W.P.G.)

**Keywords:** self-sensing, concrete, sensor, circuit, miniature, measurement, impedance, resistance

## Abstract

A vast amount of civil infrastructure is constructed using reinforced concrete, which can be susceptible to corrosion, posing significant risks. Corrosion of reinforced concrete has various causes, with chloride ingress known to be a major contributor. Monitoring this chloride ingress would allow for preventative maintenance to be less intrusive at a lower cost. Currently, chloride sensing methods are bulky and expensive, leaving the majority of concrete infrastructures unmonitored. This paper presents the design and fabrication of a miniature, low-cost device that can be embedded into concrete at various locations and depths. The device measures localized concrete resistance, correlating to the chloride ingress in the concrete using equations listed in this paper, and calculated results from two experiments are presented. The device benefits from a four-probe architecture, injecting a fixed frequency AC waveform across its outer electrodes within the cement block. Voltage across the internal electrodes is measured with a microcontroller and converted to a resistance value, communicated serially to an external computer. A final test showcases the ability of the device for three-dimensional mass deployment.

## 1. Introduction

### 1.1. NDT Methods

There exist multiple methods to sense corrosion development in reinforcement concrete. These methods can be categorized into two key groups, destructive and non-destructive. Destructive testing (DT) methods require the concrete sample to be damaged to evaluate its properties. Non-destructive testing (NDT) methods evaluate the properties of the concrete sample without causing damage to the sample.

This paper focuses on NDT methods, as these methods have shown capability for fast and reliable structural health diagnosis, with several reported approaches utilizing reinforced concrete structure [1,2,3,4,5]. Commonly, passive sensors are being used to perform measurements. These sensors use radio frequency resonance for power and communication [6,7,8,9]. Most of these sensors utilize an inductive-capacitive tank to improve the sensing mechanism. A downside is that exposure promotes a reaction with time, making the unit unstable over prolonged periods.

Other methods include the galvanostatic pulse method [10,11], polarization resistance measurement method [12,13], Wenner method of impedance measurement and ground penetrating radar (GPR) [14,15]. The Wenner method measures corroded steel reinforcement bars at the concrete surface, where corrosion is identified using low frequency alternating current (AC) waveforms injected across the steel reinforcement and calculated using Faraday’s Laws of electrolysis. An increase in corrosion produces a reduction in impedance over time [16].

Another NDT method maps direct current (DC) electrical resistivity using embedded sensors [17]. It is based on the property of materials to oppose the flow of electrical current due to the nature of the medium, its porosity and the electrolyte content [17]. It is not advised to measure concrete resistance through injecting DC into the sensing block, as this will not result in accurate resistance readings, due to polarization effects within the concrete matrix [18]. As concrete is not a purely resistive medium, utilizing a DC measurement will also induce a reactive electrical component. Injecting an AC signal into concrete reduces reactance and is the preferred method for measuring the resistive component of concrete. Adding functional fillers near their percolation concentration can improve the signal-to-noise ratio (SNR) [19] (see Section 2.1 for further details).

Prior to this work, a popular method used to provide measurements at multiple depths was GPR. This method induces radar waves to measure reflected waves off surfaces where there is a change in density/dielectric. This technique uses longer wavelengths as depth increases. GPR is costly, requires sophisticated data analysis and only provides localized monitoring. Internal reflections from the concrete can induce a poor reading and increase SNR as depth increases.

Another method to perform measurements at multiple depths is to use ultrasonic sensors to image the internal structure of concrete. Ultrasonic testing induces high frequency sound waves into the medium and measures reflected waves at the surface of the sensor [20,21,22]. This method has been used with multi-sensor arrangements. Due to the shorter wavelengths than GPR, it is limited to shallow depth measurements.

The goal of this study was to develop a miniature, multi-electrode, non-destructive embedded sensor to monitor electrical resistivity of concrete by means of measurements at multiple depths at a low cost [23]. In this study, we selected concrete polarization resistivity measurement as a suitable method of infrastructure health monitoring, as it is accurate, non-destructive and suitable for miniaturization [24,25,26]. Research has found that the electrical resistivity of concrete is an effective parameter to evaluate the risk of concrete corrosion [27].

### 1.2. Embedded Devices

Previous attempts to develop low-power embedded devices for sensing concrete corrosion have been reported in the literature. From these attempts, it was found that most embedded sensors do not optimize peak power operation. The DC electrical resistivity method uses embedded sensors relying on injecting DC current to measure resistance. Peak power consumption may not be optimized, as the embedded sensor measures a higher resistance than an AC injecting embedded sensor, therefore requiring higher current injection. Embedded wireless sensors require an inductively coupled external power supply connection, and they have been reported as consuming as high as 150 mA of current at 12 V during peak power operation [28].

This paper presents an effective, low-cost, miniature, resistance measurement device for the detection of concrete corrosion, utilizing a four-probe sensing technique [29], with a highly stable, efficient, fixed frequency AC waveform for injecting current across the external probes. The internal probes monitor and sense the voltage, and an internal algorithm calculates the effective resistance of the sample under test. Peak current consumption of 8 mA from a 3.3-V source effectively shows the device’s competitively low peak power consumption during operation, allowing multiple sensors to be embedded off a single power supply. This opens the potential for a three-dimensional measurement of concrete on different axes running at peak power for continuous chloride ingress monitoring, showing the novelty of the device.

**Figure 1:**
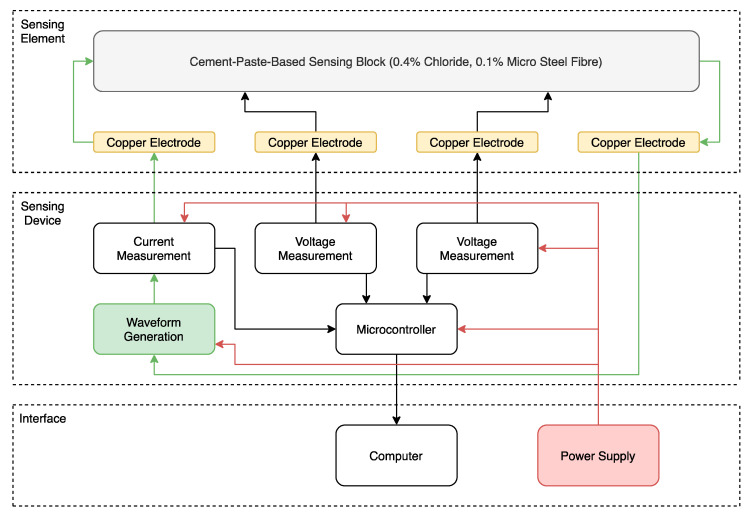
Internal components and system architecture of the concrete resistance measurement device.

The device developed to detect concrete corrosion includes the following key components: sensing element, sensing device and interface (see Figure 1). Each component performs a specific role in the measurement of the resistance of concrete and is described in the following sections.

### 1.3. Previous System

The proposed sensing device is an improved iteration of our previously developed sensing device [30] with a complete redesign of hardware and software. This includes a new waveform generation method, analog measurement method and external interfacing, with two revisions developed. Improving the sensing device was done to further lower power consumption and size of the device. The previous iteration consumed 25 mA peak current at 3.3-V supply voltage, due to the microcontroller generating the 100 kHz waveform. The internal clock speed of the microcontroller was continuously running at 32 MHz to generate this waveform. Components used on this iteration were large in size, with surface mount components selected for the new iteration to reduce overall size for embedding into concrete. The previous iteration provided clean results successfully emulating the Keysight E4980AL; however, it was observed to have slight voltage deviation after long periods of time. For this reason, developing a clean waveform generation and measurement circuit was needed for removing any DC offset the circuit might be inducing.

The use of a sinusoidal/triangular waveform was observed to provide little measurement difference between manufactured devices as compared to a square waveform. Thus, results between devices are more closely related. Different waveforms injected across the block were tested to show their validity, with square, ramp and sinusoidal providing results to measure resistance [30].

## 2. Sensing Element

Measuring the resistance of concrete using this device requires a novel composition of a cement paste. The pre-made device is intended to be embedded in new reinforced concrete infrastructure during construction. Measuring local resistance near the device would determine the surrounding concrete condition. A higher number of sensing devices would result in higher resolution of surrounding concrete condition.

For this study, the cement paste was formed into a sensing block where the electronic circuitry in the sensing device can determine the electrical resistivity, which can be correlated to chloride ingress and other electrolytes. This is shown in the Section 4, where resistivity and chloride ingress are calculated from the sensing devices resistance measurement.

### 2.1. Cement Chemistry

**Figure 2:**
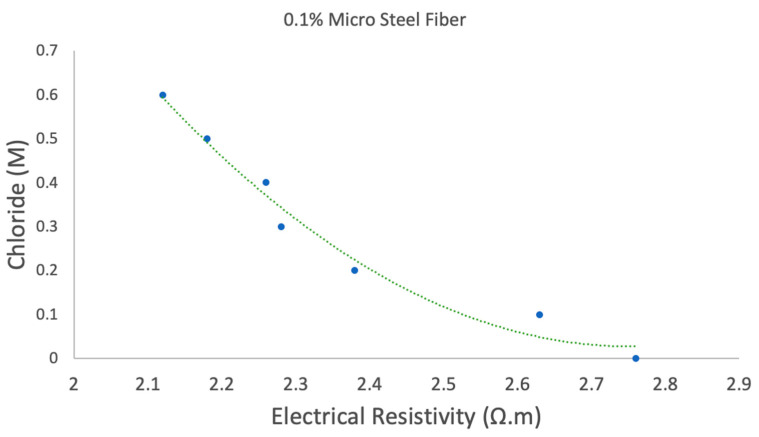
Relationship of electrical resistivity and chloride ingress containing 0.1% micro steel fiber.

The electrical resistivity of a material, represented by rho (*ρ*), is a measure of how strongly a material opposes the flow of electrical current. The range of electrical resistivity of concrete depends strongly on the concrete moisture content, with wet concrete behaving as a semiconductor with a resistivity in the range of 10^4^ Ω·cm. Dry concrete behaves as an insulator with a resistivity of 10^11^ Ω·cm [31]. Therefore, a conductive filler is a necessary ingredient for fabricating this sensor to overcome the high resistivity of concrete [32]. Adding conductive filler reduces the percolation concentration, which is the lowest concentration of filler at which insulating material is converted to conductive material. Several conductive fillers have been investigated including carbon nanotubes, carbon fiber and graphite, however micro steel fibers were chosen for their outstanding properties, including higher abrasion resistance; resistance to micro crack propagation; improvements to the tensile and flexural strength by 120% and 25%, respectively, from adding 0.53% of steel fiber (60-µm diameter and 5-mm length); non-hazardous to work with; relatively cheap (around $0.1 per gram); provides low electrical resistivity; and enhances its sensitivity to the presence of chloride [33,34,35,36]. The micro steel fibers can also be used to evaluate stress, strain and crack propagation or damage of the blocks [37,38,39,40,41]. Functional fillers such as micro steel fiber can reduce the resistivity to 200 Ω·cm and thus aid resistance measurements and improve SNR. One requirement of the device is be able to sense chloride presence in the pore water of the paste. The relationship of chloride ingress to electrical resistivity of cement paste containing 0.1 wt% micro steel fibers is presented in Figure 2.

Using Equation (1), we can relate the electrical resistivity of the cement paste to chloride ingress.

(1) $$\left[Cl^{-}\right]=1.4656\rho^{2}-8.0614\rho +11.112\;Cl=Chloride\;Concentration\;\rho =Electrical\;Resistivity$$

Relating electrical resistivity to the device’s resistance measurement is done using Equation (2).

(2) $$\rho =R\frac{A}{l}\;\rho =Electrical\;Resistivity\;R=Resistance\;A=Cross\;Sectional\;Area\;of\;Piece\;l=Length\;of\;Piece$$

where we extend the use of embedded sensors to determine chloride ingress in concrete to provide an indication of chloride ingress to the reinforcement, and thus when the onset of depassivation to the steel reinforcement [42] may occur. Therefore, continuously monitoring the device will reveal the occurrence of chloride ingress, allowing for remediation action to be taken.

### 2.2. Cement-Paste-Based Sensing Block

**Figure 3:**
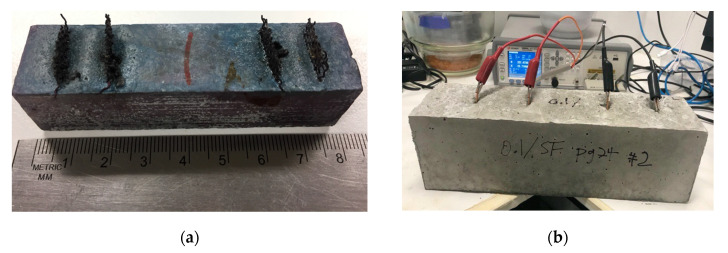
Cement paste-based sensing blocks: (**a**) Size 1; and (**b**) Size 2.

Multiple samples of cement-paste-based sensing blocks were constructed in two different sizes. Size 1 measured 80 mm × 20 mm × 20 mm and Size 2 measured 100 mm × 100 mm × 375 mm. These samples were exposed to 0.4% chloride ingress with 0.1% micro steel fiber. Exposing to 0.4% chloride is a method used for chloride exposure in previous research projects [43]. Size 1 was allocated for use in aiding the development of the sensing device shown in Figure 3a, and Size 2 was allocated for the use in testing of the sensing device shown in Figure 3b. Sizes 1 and 2 utilize copper electrodes embedded into the sensing-blocks spaced 15 mm apart on the two most outer probes and 40 mm between inner probes.

### 2.3. Resistance Measurement

An AC waveform must be applied to the sensing blocks to enable an accurate reading of resistance. With a high enough AC waveform frequency, the reactance can be minimized from the impedance readings, and the true resistance of the sensing block is conveyed. For this reason, the Keysight E4980AL LCR meter [44] was used as an AC waveform reference to verify that the developed sensing device is performing accurately. Calibrating the sensing device with the Keysight LCR meter also means that proper correction factors can be applied for an accurate reading.

Measuring the capacitance and inductance of the sensing block was done using an Agilent U1731B [45] to determine the reactance. The condition of the cement blocks while conducting this measurement was different to when first conducted including the condition of the electrodes. At 120 Hz, the block measures 5.7 nF of capacitance and 20.42 H of inductance. At 1 kHz, the block measures 330 pF of capacitance and 805 mH of inductance. The capacitive reactance is determined by:

(3) $$Xc=\frac{1}{2*\pi *f*C}\;Xc=Capacitive\;Reactance\;f=Frequency\;C=Capacitance$$

The inductive reactance is determined by:

(4) Xl=2∗π∗f∗LXl=Inductive Reactancef=FrequencyL=Inductance

The total reactance is determined by:

(5) X=Xl−XcX=ReactanceXc=Capacitive ReactanceXl=Inductive Reactance

**Figure 4:**
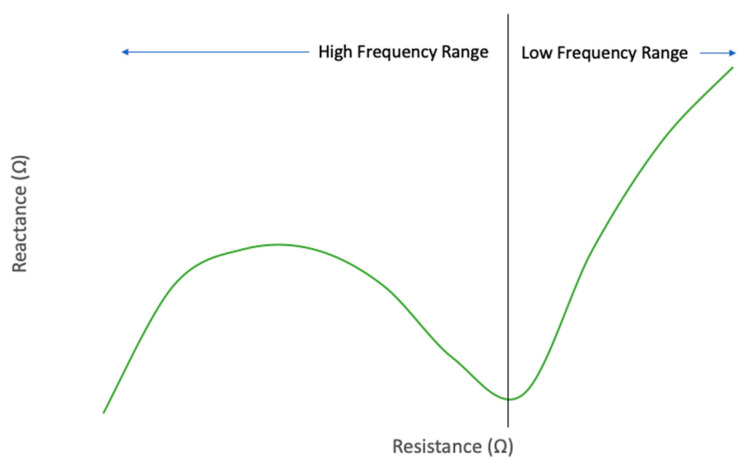
Impedance of concrete.

Using these equations, the reactance of the cement block resulted in 216.6 kΩ at 120 Hz and 476.9 kΩ at 1 kHz. This increase in impedance declines with frequencies higher than 10 kHz, as shown in Figure 4. For this reason, 100 kHz was the selected frequency [46]. This frequency balances low impedance and low power, as an increase in waveform frequency can result in higher power consumption and circuit complexity.

## 3. Sensing Device

**Figure 5:**
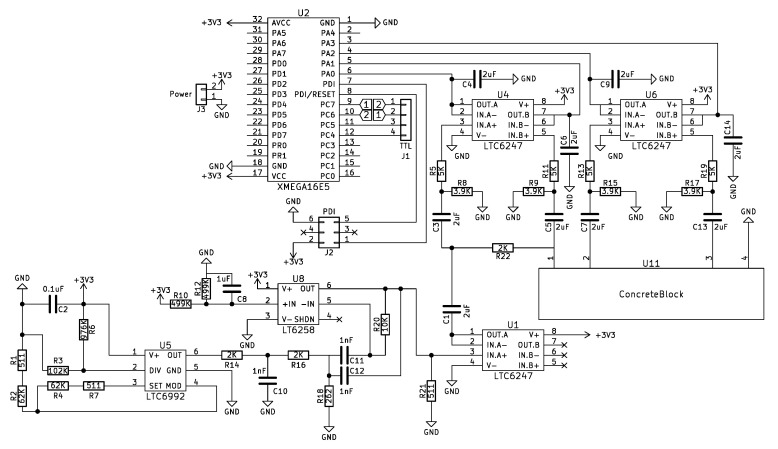
Electronics schematics of the sensing device.

An accurate device that can measure within ±10 Ω of a reference value with minimal deviation over time is presented. This device is low power, low cost and has two form factors: small and extra small footprints. These devices can be embedded into concrete with appropriate ingress protection and successfully measure resistance where calculation for resistivity and chloride ingress can be achieved, as shown in the Section 4. Figure 5 presents the electronic schematic.

### 3.1. Waveform Generation

**Figure 6:**
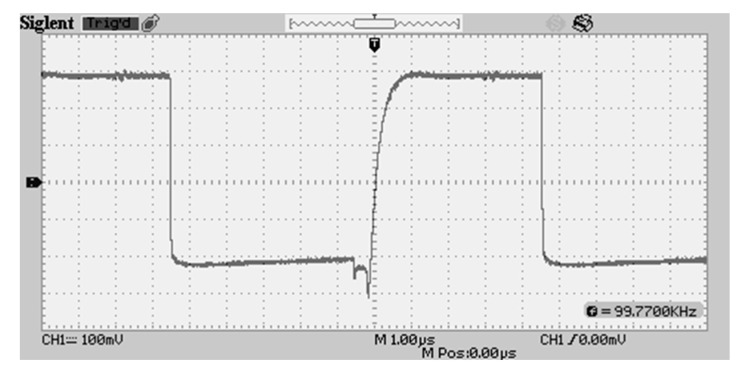
Previous waveform delivered to the sensing element.

Previous methods of waveform generation incorporated a constant current DC voltage supply injected into a transistor switching at 100 kHz, fed into a capacitor, emulating a square wave generator while consuming low power. The waveform fed across the cement block by the previous device is pictured in Figure 6. This method is simplistic in design and could be implemented with a minimum number of components; however, this method did not provide a clean waveform for feeding into the sensing block over an extended period of time. For the waveform to be effective after prolonged periods, very low DC offset values from the waveform are required, meaning positive and negative cycles of the waveform must be identically mirrored. Such a requirement is very complex to achieve because many electronic components output a DC quiescent offset current, leading to charge retention within the cement block. Thus, the cement block requires a highly purified, zero-offset AC waveform to be fed into it while maintaining low cost, low power and a small footprint, but with large sample volume (relative to footprint). In addition, the design should be able to operate in an array configuration within concrete masses.

New electrical components were required to redesign a new waveform generator that provides a highly purified AC waveform. This redesigned circuit begins with an LTC6992, a voltage-controlled pulse width modulator generator. The modulated output is set to a 50% duty cycle with a frequency of 100 kHz using passive components to select the frequency with Equation (6).

(6) $$F=\frac{1\;\mathrm{MHz}}{N_{DIV}}*\frac{50\;k\Omega }{R_{SET}}\;F=Frequency\;N_{DIV}=Internal\;Frequency\;Divider\;R_{SET}=Setting\;Resistor$$

The output waveform from the PWM generator is a square wave in the positive domain with a 3 V DC offset (supply voltage). This waveform is fed into a custom, active band-pass filter, specifically a narrow bandpass filter tuned to a center resonant frequency of 100 kHz. The resonant frequency is found using Equation (7) where the −3 dB low pass frequency and −3 dB high pass frequency multiplied and square rooted determines the resonant frequency.

(7) $$f_{r}=\sqrt{f_{l}*f_{h}}\;f_{r}=Resonant\;Frequency\;f_{l}=Low\;Pass\;Frequency\;f_{h}=High\;Pass\;Frequency$$

The active band pass filter is a second-order Butterworth filter with a 12 dB/octave roll off at its high and low pass filters. The Q-factor determines the overall width of the actual pass band between upper and lower −3-dB corner points with a Q-factor of 2, leaving headroom for tolerances in passive components to not meet the 100-kHz center frequency. The active component of the bandpass filter is the LTC6258 operational amplifier with passive components determined from Equation (8), with *R*_1_ and *R*_2_ determined from the Q-factor.

(8) $$f_{r}=\frac{1}{2*\pi *C*\sqrt{R_{2}*R_{1}}}\;f_{r}=Resonant\;Frequency\;C=Capacitor\;R_{n}=Resistor$$

The active band pass filter converts the square wave into a purified sinusoidal/triangular waveform with a 3 V DC offset. The output from this bandpass filter is regulated from active circuitry, meaning a resistor is needed to provide a load on the output. This DC offset sinusoidal/triangular waveform is fed into a unity gain buffer operational amplifier. The chosen buffer amplifier is the LTC6247. The high impedance input of the operational amplifier ensures very little current is drawn from the main waveform circuitry, while also ensuring a correct voltage drop across the cement block is achieved, acting as an unregulated voltage output. The final stage of the waveform generator is to remove the DC offset. This is achieved from an AC coupling capacitor tuned to the resonant frequency of the circuit, in this case 100 kHz, with a value of 2 µF achieved using Equation (9).

(9) $$C=\frac{1}{2*\pi *f*Xc}\;C=Capacitance\;f=Frequency\;X_{c}=Capacitive\;Reactance$$

**Figure 7:**
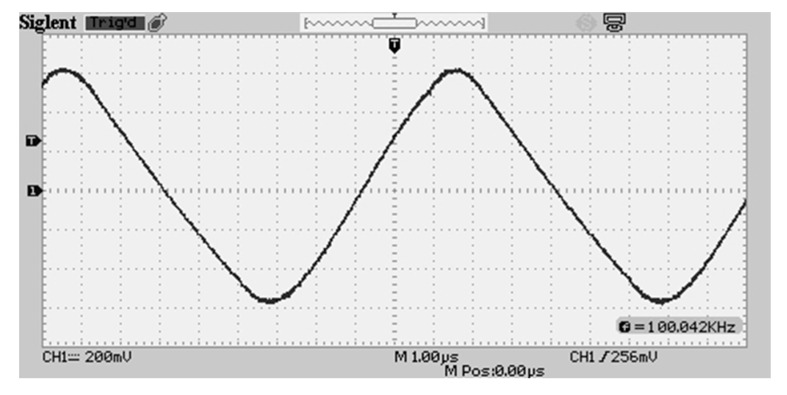
New waveform delivered to the sensing element.

Total circuit power draw from the waveform generator is only 0.42 mW with only 1 mA required at a 1.2-V peak-to-peak voltage. Reducing the peak-to-peak voltage will reduce the power consumption further. The waveform is sinusoidal/triangular in nature with very little DC offset. The new waveform fed across the cement block is pictured in Figure 7.

**Figure 8:**
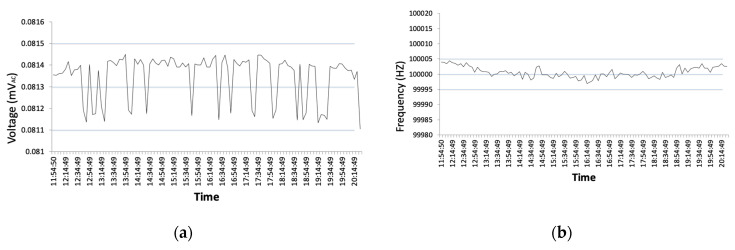
Test conducted over 8 h using HP 34970A: (**a**) AC voltage deviation; and (**b**) frequency deviation.

Running the new waveform generator in temperature conditions between 18–25 °C, over 8 h, the frequency deviation was within ±5 Hz tolerance at 100 kHz. Running the waveform generator through a dummy resistor showed a voltage deviation of ±0.2 mVAC after the same extended period of time. Samples were taken for these tests with the HP 34970A data acquisition unit [47], taking over 100 samples in 15-min intervals. Results from these tests are shown in Figure 8a,b.

### 3.2. Cement Block

The electrical properties of the cement block do not behave as a simple resistor. Small conductive material and four metal probes embedded into the cement block have a capacitive effect when a current is injected. This means any DC injected into the cement block will lead to charge retention, creating a DC offset to near supply level voltage, inducing reactance.

### 3.3. Measurement System

The measurement technique for the sensing device utilizes a four-probe technique, allowing conversion to a resistance value. The Atmel microcontroller ATxmega16E5 was chosen as the central processing unit, with an in-built 12-bit analog-to-digital converter (ADC) reading a high-resolution analog voltage from the cement blocks. On the front end of the ADC is a unity gain buffer operational amplifier. The chosen buffer amplifier is the LTC6247. The high impedance input of the unity gain buffer ensures no current from the waveform generator is to be fed into the microcontroller. It also serves the purpose of half wave rectifying the AC waveform. Traditionally, this is done with a diode in series with the ADC, however diodes contain a forward voltage as high as 0.7 V when the measured voltage could be as little as 0.01 V depending on the cement chloride ingress. Using the unity gain buffer ensures no voltage drop, as it is an active component, while consuming minimal power. Two LTC6247 operational amplifiers were used, both containing two channels. One was used for voltage measurement across the block and another was used for a resistor in series with the cement block to measure current across the block. All front ends of the operational amplifiers are AC coupled with a capacitor using Equation (4). A voltage divider on the front-end of the operational amplifiers is also used with a high resistance value as to not consume too much power, while providing a cleaner reading on the input of the operational amplifier. The four outputs of these operational amplifiers are fed to the multiplexed input of the ADC converter of the microcontroller. A capacitor can be added to the output of the operational amplifiers as to improve signal quality by means of storing charge converting the signal into a DC output; however, they also increase the power consumption, making them optional when more resolution is needed.

### 3.4. Software

Software used in the sensing device begins with an initialization of peripherals and ports. The program works by continuously sampling the multiplexed input of the ADC. The voltages read by the ADC are an AC waveform, only reading the positive domain. For this reason, the program takes multiple samples and collects the highest sample looking for the peak of the waveform. Once the program detects the peak of the waveform, the root-mean-square (RMS) value can be calculated by multiplying the voltage by 0.707. The first two analog inputs are across the series resistor where the voltage is read by the program. These two RMS voltages are subtracted from each other and calculated using Equation (10), where resistance is of a known fixed value.

(10) $$I=\frac{V1-V2}{R}\;I=Electrical\;Current\;through\;Resistor\;V_{n}=Sensed\;Voltage\;through\;Resistor\;R=Resistance$$

After the program calculates the current, the last two analog inputs are read from the cement block, where the program calculates the resistance of the cement block using Equation (11).

(11) $$R=\frac{V1-V2}{I}\;R=Resistance\;of\;Cement\;Block\;V_{n}=Sensed\;Voltage\;through\;Cement\;Block\;I=Electrical\;Current\;through\;Cement\;Block$$

**Figure 9:**
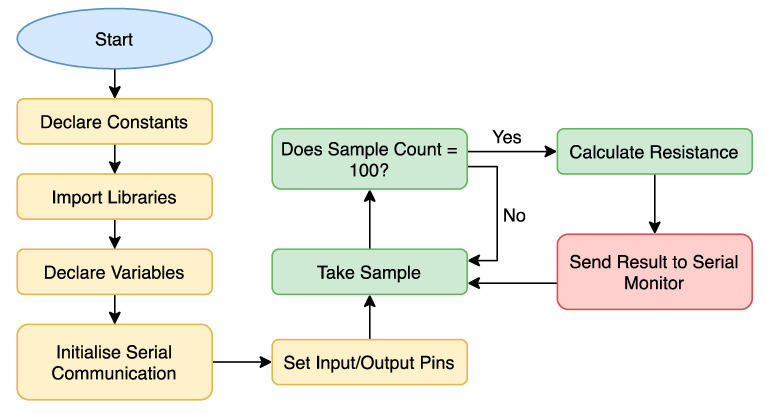
Software flowchart run on the microcontroller.

After the program calculates the resistance of the cement block, the results are transmitted to a computer to be displayed to the user, at a sampling rate of 1 s. The flowchart in Figure 9 displays the main modules of the C program uploaded to the ATxmega16E5 microcontroller.

### 3.5. Interfacing

The communication method for the sensing device is via a wired interface. The communication protocols are either transistor–transistor logic or serial peripheral interface, selected in the program. A USB-to-TTL converter is used for communicating to the computer, also providing a 3.3-V power supply to the circuit. This USB-to-TTL converter uses a CP2102. SPI can be used for communication to multiple sensing devices via the four-wire interface only selectable from the small footprint variant.

### 3.6. Revisions

**Figure 10:**
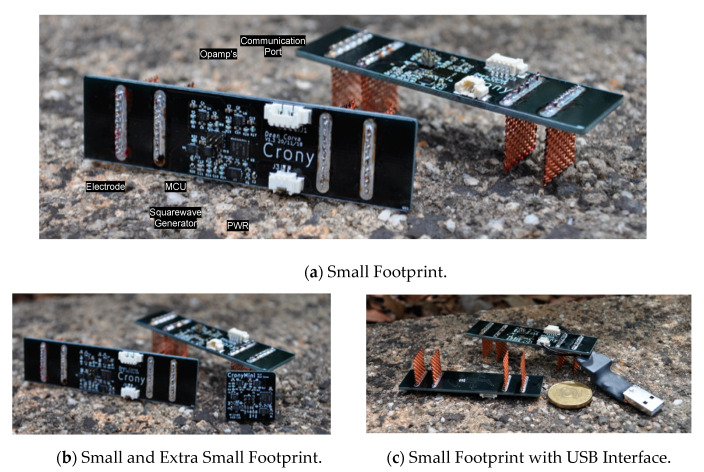
Two device form factors of small and extra small for embedding into concrete: (**a**) small revision dimensions are 20 mm × 20 mm × 80 mm; (**b**) extra small revision dimensions are 3 mm × 15 mm × 15 mm. (**c**) A standard USB A connector and an Australian Dollar, measuring 25 mm, are shown for comparison.

Two revisions of the sensing device were fabricated. These revisions are of two form factors, small (see Figure 10a–c) and extra small (see Figure 10b). These footprints are similar in size to large aggregate of concrete, with potential to be made wireless through communications to nearby sensors. The first revision measures 20 mm × 20 mm × 80 mm and includes external electrodes for the four-probe sensing included in the printed circuit board. This revision can be configured to run USB output or SPI output via external wires. The second revision is a smaller version measuring 3 mm × 15 mm × 15 mm and includes a connector for connecting external probes for the four-probe sensing. This revision includes a USB connector on the back.

### 3.7. Complete Concrete-Based Sensing Device

A key aim of this device was that the sensing devices could be integrated into the concrete itself. For this reason, protecting the circuitry was of high importance. A 3D printed enclosure was fabricated and adhered onto the PCB itself, with external probes protruding out the enclosure and one cable for interfacing. After testing the sensing device on multiple cement blocks sized 80 mm × 20 mm × 20 mm, the sensing device was implanted into a concrete block measuring 100 mm × 100 mm × 375 mm. Total power consumption of the sensing device running at full sampling rate is 26 mW, drawing 8 mA of current at 3.3-V supply voltage. The device will only operate when connected through USB.

## 4. Results and Discussion

Using the Keysight E4980AL LCR meter to compare the resistance readings of five cement-paste-based sensing blocks to the manufactured device was done to ensure the device is within ±10 Ω. Blocks 1–5 are of Size 1 dimensions. The results of the testing Blocks 1–5 are displayed in Table 1.

Equation (2) was used to calculate the resistivity of the blocks to find chloride ingress. The results of calculating resistivity are displayed in Table 2.

Calculating the chloride ingress of the blocks was then done using Equation (1), and the results are displayed in Table 3.

These results show the sensing device performed within the expected region of results. This demonstrates the effective region of resistance is 400–550 Ω for detecting chloride presence, as this is 2–2.74 Ω·m of resistivity, which correlates to minimal chloride presence to above 0.6% chloride presence. This justifies the ±10 Ω tolerance range, as chloride presence produces a significantly larger signal.

**Figure 11:**
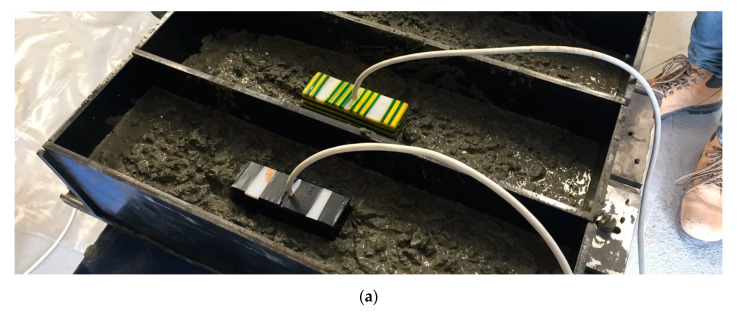

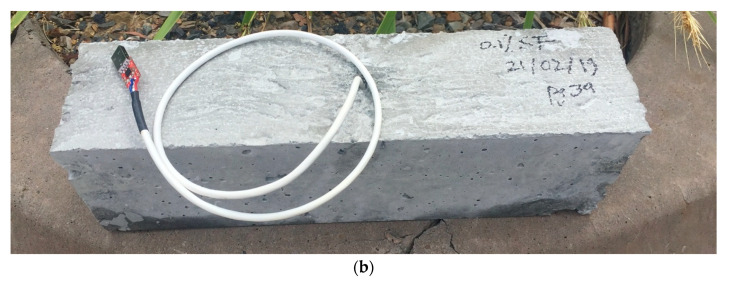
Sensing device embedded into sensing element: (**a**) sensing device pre-concrete pouring; and (**b**) sensing device post-concrete pouring.

Embedding two devices into large cement-paste-based sensing blocks of Size 2 dimensions was completed to show effectiveness even when embedded. Figure 11a,b shows the embedding of the device. Measurement results were taken when the sensing blocks were submerged in a tank of CaOH liquid solution to aid the curing process and prevent the added chloride ions in the cement-paste-based sensing blocks from leaching out. The results from embedding the device are shown in Table 4.

The calculated resistivity of the blocks is shown in Table 5.

The calculated chloride ingress of the blocks is shown in Table 6.

**Figure 12:**
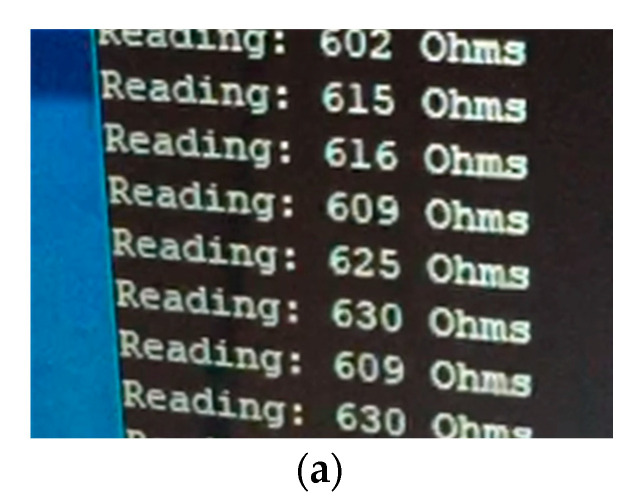

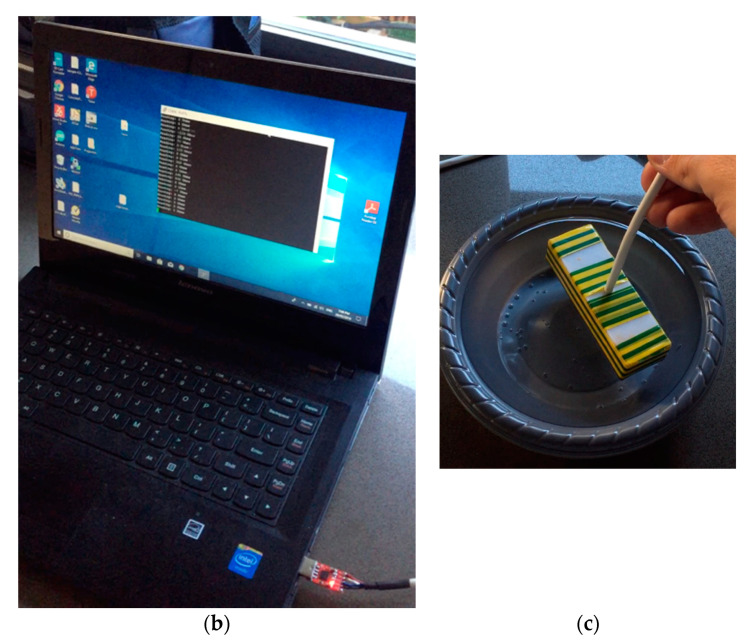
Sensing device tested in water and results displayed on laptop: (**a**) close-up of resistance displayed on laptop; (**b**) resistance displayed on laptop; and (**c**) sensing device in water.

These results show a clean and effective solution for the measurement of chloride ingress, ready for the implementation into the novel composition of cement paste. Testing the device in water connected to a computer is pictured in Figure 12a–c. The test was conducted using basic tap water and demonstrated the sensing devices ability to measure non-concrete materials. The results from testing five times in water are displayed in Table 7 with results averaged after 10 samples.

**Figure 13:**
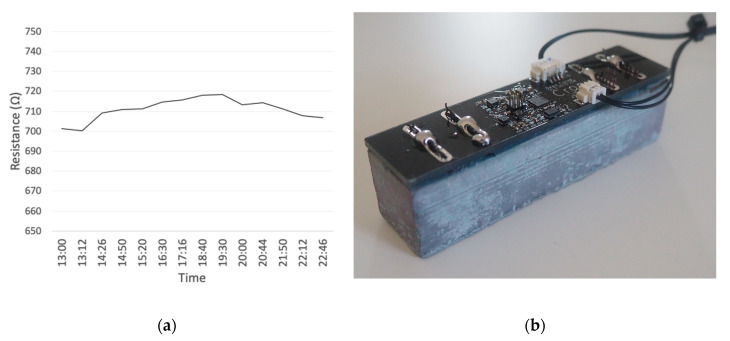
Sensing device mounted to sensing block, run for 10 h to show deviation: (**a**) voltage deviation over 10 h; and (**b**) sensing device setup.

Running the sensing device on one of the small blocks for 10 h shows the voltage deviation over time for continuous monitoring. The higher resistance values are due to leaving the blocks out for an extended period from when the blocks were first manufactured. This is due to there being no water ionic conductivity, required for detecting chloride ingress. The results from this test are displayed in Figure 13a. Figure 13b shows the setup of the device with the sensing element mounted directly to the sensing device. The system was powered and communicated to a computer for 10 h.

The results from this test show that the temperature change throughout the day increased the offset observed in the results. The reduction of the resistance towards the end of the test shows that the device has the ability not to induce DC offsets by using the new waveform.

Further future work can be done on this project including further miniaturization of the PCB and integration of an RFID induction coupler to power the system and transmit data wirelessly. Power consumption from these revisions is within the capabilities of RFID induction coupling.

## 5. Conclusions

This paper demonstrates the suitability of a novel sensing device embedded into concrete to measure the electrical resistance, and by correlation the ingress of chloride in concrete pore water. Using the zero-offset AC waveform that injects current into a four-probe architecture across the outer electrodes, the electrical resistance of the cement is determined. Chloride ingress in the local pore water can be estimated to enhance early detection of the onset of reinforcement corrosion. This was tested with five cement blocks and compared with the Keysight E4980AL LCR meter, shown to be operating within ±10 Ω of specifications. The low power operation of the device allows for multiple different power and communications options, with RFID induction or hardwired connection, as demonstrated in the study. The small form factor of the device means embedding it into concrete will only require a small footprint, allowing for multiple sensors to be embedded at different depths, meaning a resistance matrix can be determined, showing chloride ingress in a three-dimensional space. By embedding an array of sensors within reinforced concrete, the sensors can be used to improve management of new civil infrastructure in aggressive environments.

## Figures and Tables

**Table 1:** Experiment 1 resistance measurement results in Ω.

Resistance Measurement	Keysight E4980AL	Sensing Device
Block 1	325	330
Block 2	278	271
Block 3	439	440
Block 4	433	430
Block 5	477	475

**Table 2:** Experiment 1 resistivity calculation results in Ω·m.

Resistivity Calculation	Keysight E4980AL	Sensing Device
Block 1	1.63	1.65
Block 2	1.38	1.36
Block 3	2.2	2.2
Block 4	2.17	2.15
Block 5	2.39	2.38

**Table 3:** Experiment 1 chloride calculation results in M.

Chloride Calculation	Keysight E4980AL	Sensing Device
Block 1	1.87	1.8
Block 2	2.78	2.86
Block 3	0.47	0.47
Block 4	0.52	0.55
Block 5	0.22	0.23

**Table 4:** Experiment 2 resistance measurement results in Ω.

Resistance Measurement	Sensing Device
Block 1	321
Block 2	330

**Table 5:** Experiment 2 resistivity calculation results in Ω·m.

Resistivity Calculation	Sensing Device
Block 1	1.6
Block 2	1.65

**Table 6:** Experiment 2 chloride calculation results in M.

Chloride Calculation	Sensing Device
Block 1	1.97
Block 2	1.8

**Table 7:** Experiment 3 resistance measurement results in Ω.

Resistance Measurement	Sensing Device
Sample 1	624
Sample 2	622
Sample 3	628
Sample 4	614
Sample 5	631
